# Successful living-donor liver transplantation after treatment of sinus aspergillosis by endoscopic mycetoma removal and sinus drainage

**DOI:** 10.1186/s40792-015-0029-1

**Published:** 2015-03-12

**Authors:** Norimitsu Okui, Hiroaki Shiba, Shigeki Wakiyama, Yasuro Futagawa, Yuichi Ishida, Katsuhiko Yanaga

**Affiliations:** Department of Surgery, The Jikei University School of Medicine, 3-25-8, Nishi-Shinbashi, Minato-ku, 105-8461 Tokyo Japan

**Keywords:** *Aspergillus*, Aspergillosis, Liver transplantation, PBC

## Abstract

A 47-year-old female was admitted to our hospital for treatment of end-stage liver disease due to primary biliary cirrhosis. Preoperative routine nasal sinus magnetic resonance imaging revealed diffuse inflammatory mucosal hyperplasia of the right maxillary sinus and mycetoma without invasive fungal sinusitis. *Aspergillus* antigen was positive. With a diagnosis of sinus aspergillosis, endoscopic sinus drainage and removal of mycetoma were performed. After endoscopic treatment, the right maxillary sinus was irrigated using amphotericin B for 2 weeks and then treated by iodine with gentamicin and ketoconazole for 6 weeks. At 1 month after endoscopic treatment, the mycetoma had disappeared. At 3 months after the endoscopic treatment, the patient underwent living-donor liver transplantation using the left and caudate lobe of her daughter. The patient made a satisfactory recovery and was discharged on 19 days after transplant. As of 44 months after transplant, she remains well without recurrence of aspergillosis.

## Background

In immunosuppressed patients including organ transplant recipients, infection with *Aspergillus* species results in excess mortality rates with the range from 58% to 87% [1,2]. Therefore, pre-transplant evaluation of infectious complications and complete elimination of infectious focuses are important. We report a liver transplant recipient after treatment of sinus aspergillosis by endoscopic mycetoma removal and sinus drainage. To the best of our knowledge, such a case has not been reported in the English literature.

## Case presentation

**Figure 1:**
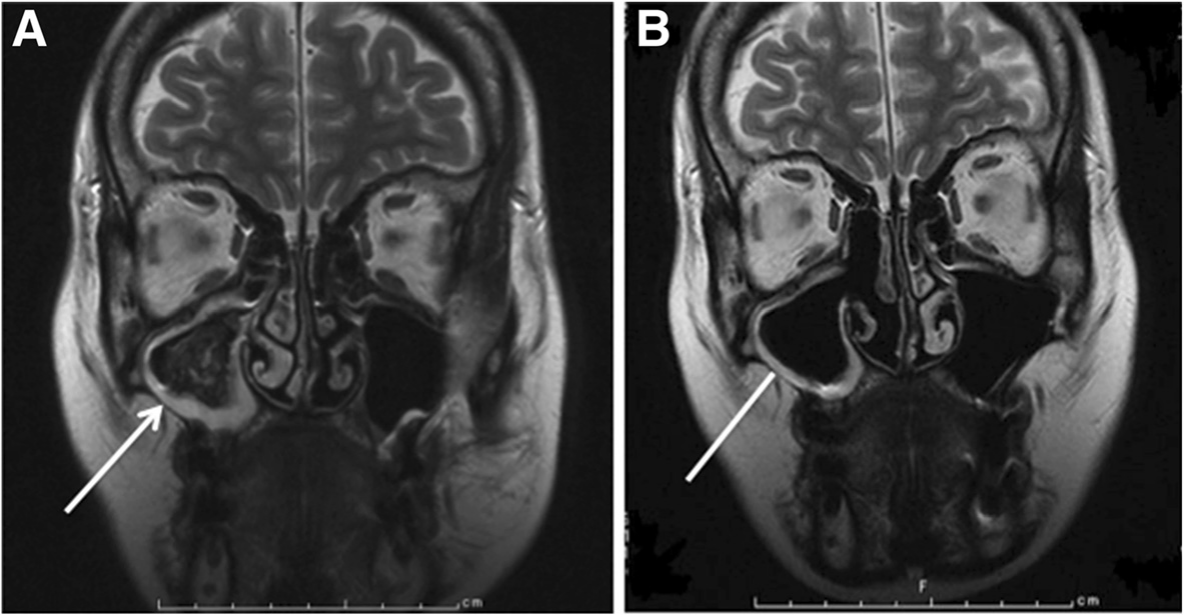
**Pre- and postoperative nasal sinus magnetic resonance images are shown.** Preoperative routine nasal sinus magnetic resonance image revealed diffuse inflammatory mucosal hyperplasia of the right maxillary sinus, and a mycetoma without invasive fungal sinusitis in T2-weighted image (**(A)**, arrow). At 1 month after endoscopic treatment, diffuse inflammatory mucosal hyperplasia of the right maxillary sinus improved and mycetoma disappeared (**(B)**, arrowhead).

**Figure 2:**
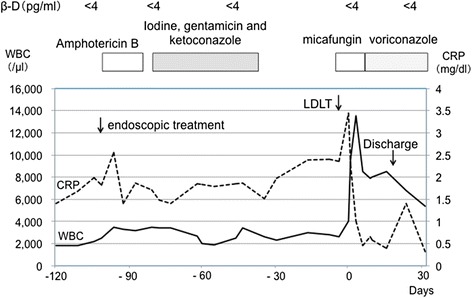
**Before endoscopic treatment, C-reactive protein was 2.0 mg/dl and beta-D glucan was less than 4 pg/ml.** After endoscopic treatment, the right maxillary sinus was irrigated using amphotericin B for 2 weeks and then treated by iodine with gentamicin and ketoconazole for 6 weeks. At 3 months after the endoscopic treatment, the patient underwent living-donor liver transplantation and was discharged on 19 days after transplantation.

A 47-year-old female was admitted to our hospital for treatment of end-stage liver disease (ESLD) due to primary biliary cirrhosis (PBC). The patient had undergone endoscopic treatment for ruptured esophageal varices in 2003 and had hypothyroidism and Sjögren’s syndrome. The patient’s model for end-stage liver disease score was 19, and the Mayo risk score was 9.18. Preoperative routine nasal sinus magnetic resonance image revealed diffuse inflammatory mucosal hyperplasia of the right maxillary sinus and mycetoma without invasive fungal sinusitis (Figure 1A). Histological examination of mycetoma revealed aspergillosis. Before treatment of the sinus aspergillosis, laboratory investigations included C-reactive protein of 2.0 mg/dl, beta-D glucan less than 4 pg/ml, and positive *Aspergillus* antigen (Figure 2). With a diagnosis of sinus aspergillosis, endoscopic sinus drainage and removal of the mycetoma were performed. The pathological diagnosis was aspergillosis of the right maxillary sinus. After endoscopic treatment, the right maxillary sinus was irrigated using amphotericin B for 2 weeks and then treated with gentamicin and ketoconazole after flushing using iodine for 6 weeks. At 1 month after endoscopic treatment, diffuse inflammatory mucosal hyperplasia of the right maxillary sinus improved and the mycetoma had disappeared (Figure 1B), and *Aspergillus* had become negative in maxillary culture. Before liver transplantation, beta-D glucan was less than 4 pg/ml; however, *Aspergillus* antigen remained positive, and C-reactive protein ranged from 1.4 to 2.4 mg/dl (Figure 2). At 3 months after the endoscopic treatment, the patient underwent living-donor liver transplantation (LDLT) using the left and caudate lobe of her daughter. Tacrolimus and steroids were used for initial immuno-suppression. For postoperative antifungal prophylaxis, micafungin was used for 10 days and, thereafter, voriconazole for 7 weeks. The patient made a satisfactory recovery and was discharged on 19 days after LDLT. As of 44 months after transplant, she remains well without recurrence of aspergillosis.

## Conclusions

Advances in surgical technique, immunosuppressive agent, and perioperative management have improved therapeutic outcome of liver transplantation. Fungal infections are one of the most important infectious complications of liver transplantation, contributing significantly to both morbidity and mortality [3].

Incidence of *Aspergillus* infections has been reported in 1% to 15% of organ transplant recipient. In invasive aspergillosis, the reported mortality rate ranges from 74% to 92% [4]. Therefore, postoperative antifungal prophylaxis and treatment are important to improve therapeutic outcome of liver transplantation [5]. Because preoperative presence of aspergillosis may be a high risk factor for postoperative fatal aspergillosis, complete elimination of infectious lesion before liver transplantation is necessary. Endoscopic sinus surgery is the gold standard for treatment of sinus aspergillosis with normal immune patients and antifungal therapy is unnecessary [6,7]. The management of recipients with sinus noninvasive aspergillosis is not established on the United States practice guidelines for aspergillosis [8]. Therefore, during the first 10 days after liver transplantation, micafungin was used for antifungal prophylaxis due to less drug interaction with tacrolimus. Then, antifungal prophylaxis was switching from micafungin to voriconazole, which is recommended for treatment of invasive aspergillosis on the United States guideline. Tsuchiya et al. reported a case of successful LDLT in a patient with preoperative fungal liver and splenic abscesses which was treated with amphotericin B for 3 months before liver transplant [9].

Patients with ESLD and preoperative fungal infection may become possible liver transplant candidate by complete elimination of fungal infectious lesions and adequate antifungal treatment before liver transplantation.

## Consent

Written informed consent was obtained from the patient for publication of this case report and any accompanying images. A copy of the written consent is available for review by the Editor-in-Chief of this journal.

## Abbreviations

ESLD: end-stage liver disease
LDLT: living-donor liver transplantation
PBC: primary biliary cirrhosis
